# Factors Associated with Pneumonia in Patients Hospitalized with COVID-19 and the Role of Vaccination

**DOI:** 10.3390/vaccines11081342

**Published:** 2023-08-08

**Authors:** Antonella Zizza, Raffaella Sedile, Francesco Bagordo, Alessandra Panico, Marcello Guido, Tiziana Grassi, Federico Banchelli, Pierfrancesco Grima

**Affiliations:** 1 Institute of Clinical Physiology, National Research Council, 73100 Lecce, Italy; raffaella.sedile@cnr.it; 2 Department of Pharmacy-Pharmaceutical Sciences, University of Bari Aldo Moro, 70121 Bari, Italy; 3 Department of Biological and Environmental Science and Technology, University of Salento, 73100 Lecce, Italy; alessandra.panico@unisalento.it (A.P.); marcello.guido@unisalento.it (M.G.); tiziana.grassi@unisalento.it (T.G.); 4 Department of Medical and Surgical Sciences, University of Modena and Reggio Emilia, 41121 Modena, Italy; federico.banchelli@unimore.it; 5 Unit of Statistical and Methodological Support to Clinical Research, University Hospital of Modena, 41121 Modena, Italy; 6 Infectious Diseases Unit, Vito Fazzi Hospital, 73100 Lecce, Italy; pierfrancescogrima@yahoo.it

**Keywords:** COVID-19, pneumonia, vaccination, comorbidities, SARS-CoV-2

## Abstract

Patients with COVID-19 can develop different forms of the illness with more or less severe symptoms. A 2-year retrospective cohort study was conducted to evaluate the factors associated with the development of pneumonia in patients hospitalized with COVID-19 from March 2020 to February 2022. A total of 385 patients (59.0% males) with a mean age of 69.0 ± 16.0 years were included. At hospital admission, 318 patients (82.6%) reported one or more comorbidities, namely 201 (52.2%) subjects were affected by hypertension, 98 (25.5%) type 2 diabetes, 84 (21.8%) obesity, 36 (9.4%) cancer, and 14 (3.6%) suffered from kidney disease and were being treated with dialysis, and 76 (19.7%) resulted in being vaccinated with a higher prevalence of BNT162b2 vaccine (15.0%). Pneumonia was diagnosed in 276 (71.7%) patients. Multivariate regression analysis showed that pneumonia in COVID-19 patients was positively associated with type 2 diabetes (OR 1.81; 95% CI 1.00–3.27), obesity (OR 2.52; 95% CI 1.27–4.98), and negatively with hypertension (OR 0.58; 95% CI 0.35–0.96). Vaccination against SARS-CoV-2 resulted in a strongly protective factor against the development of pneumonia in COVID-19 patients (OR 0.49; 95% CI 0.28–0.85).

## 1. Introduction

Coronavirus disease 2019 (COVID-19) is an infectious illness caused by a very contagious virus, the Severe Acute Respiratory Syndrome Coronavirus 2 (SARS-CoV-2), that primarily affects respiratory epithelial cells and can cause severe lung damage [1,2].

Angiotensin-converting enzyme 2 (ACE2) has been identified as a cellular receptor capable of mediating the binding of the virus through the spike protein and allowing it to enter the cell. The rapid replication of SARS-CoV-2 in the lungs, whose cells express high levels of ACE2, can trigger an intense immune-inflammatory response, leading to the development of acute respiratory distress syndrome (ARDS) in some cases, characterized by respiratory distress associated with hypoxemia and the presence of bilateral infiltrates on chest imaging, currently considered the leading cause of death in patients with COVID-19 [2,3].

Numerous articles have attempted to summarize and delineate symptoms according to the severity of the SARS-CoV-2 disease [4,5,6]. Most COVID-19 patients present a self-limiting viral infection with cough, sore throat, headache, myalgia, fever, and other cold or flu symptoms. Therefore, most cases of COVID-19 are mild or moderate and do not require hospitalization. However, in some patients, these symptoms progress to a lower respiratory tract infection. Among patients requiring hospitalization, the most common diagnosis is pneumonia, characterized by “ground glass” pulmonary infiltrates [7], which in the most severe forms can compromise breathing and gaseous exchange up to determining acute respiratory syndrome (3%) requiring external support (oxygen or ventilation) [8].

Since 11 March 2020, when the World Health Organization (WHO) declared COVID-19 as a pandemic [9], the virus hit the world in successive waves, also due to the emergence of new variants often showing different transmissibility and severity, as well as greater antibody escape than prior variants [10]. In particular, from the end of 2020 onwards, the Alpha variant spread to a large part of the world. In 2021, it was rapidly replaced by the even more lethal Delta variant, and from December 2021 onwards, by the more contagious Omicron variant, which was then followed by the Omicron 2 and Xe sub-variants [11].

Following the identification of the SARS-CoV-2 virus and its genome, the scientific community has contributed in a very short space of time to the development of hundreds of vaccine projects [12]. A few of these new vaccines were approved due to the state of the emergency and were administered to healthcare workers by the end of 2020 in order to gain immunity against the virus [13,14].

The prevalence of infections, hospitalizations, and deaths from COVID-19 varied with the evolution of the pandemic, among and within countries and regions worldwide, depending on the variants’ transmissibility and virulence as well as the adopted level of restrictions [15]. This prompted questions about risk and protective factors for the development of different severity levels of the disease.

Based on current evidence, the considerable variability in disease susceptibility and progression was related to demographic factors, such as age, gender, and ethnicity to genetic characteristics, as well as to the presence of underlying diseases, such as cardiovascular diseases, hypertension, and chronic obstructive pulmonary disease (COPD) [16,17,18,19,20,21,22,23]. As with most diseases, subjects with comorbidities tend to be more exposed to contracting the virus but, above all, to suffer a more severe course of the disease [21].

In addition to these variables, the main risk factors for COVID-19 severity and mortality also include laboratory indices, pro-inflammatory cytokine levels, and complications [24]. Numerous markers of inflammation have been associated with the clinical severity of COVID-19; higher values of IL-6, CRP, and D-dimer have been found in severe and critical forms of COVID-19 [25]. Moreover, the partial pressure of oxygen/fraction of inspired oxygen (PaO_2_/FiO_2_) ≤ 200 mmHg, such as stated by the consensus committee, distinguishes ARDS from the more inclusive acute lung injury [26].

In contrast, healthy eating habits, COVID-19 vaccine, and atopic conditions are considered protective against the disease and may avoid progression and poor clinical outcome [27,28,29].

Retrospective analysis of COVID-19 patient data may allow for a better understanding of the pathogenesis and the factors influencing the course of the disease as well as the development of pneumonia. Our study aimed to evaluate the factors associated with pneumonia in a cohort of patients hospitalized with COVID-19 and the role of vaccination in the prevention of severe forms of the disease.

## 2. Materials and Methods

### 2.1. Study Design

A retrospective observational cohort study was conducted to evaluate the factors associated with the severe form of COVID-19. The study cohort was made up of patients hospitalized between 1 March 2020 and 28 February 2022 for COVID-19 at the “Vito Fazzi” Hospital in Lecce (Italy), the largest one in the province of Lecce (776,230 inhabitants) [30] and a reference center for the hospitalization of subjects affected by SARS-CoV-2 during the pandemic. In particular, all patients who in the reference period had made their first access to the infectious diseases operating unit (IDOU) of the “Vito Fazzi” Hospital were considered eligible (n = 390). The sample size was determined by the number of all consecutive patients accessed to the IDOU. Then, a posterior assessment of this sample size for the development of a multivariable logistic model was provided based on the events-per-variable (EPV) ratio criterion. An EPV ratio greater than 10 was considered adequate [31].

COVID-19 diagnosis was confirmed through real-time reverse transcription polymerase chain reaction (qRT-PCR) assays in nose and oropharyngeal swabs using Cobas SARS-CoV-2 qualitative assay (Roche Molecular System, Inc., Pleasanton, CA, USA) on Cobas 6800 System. At the time of hospital admission, the demographic characteristics of the patients, as well as information relating to their clinical conditions, including pneumonia and any comorbidities, as well as SARS-CoV-2 vaccination, including the type of vaccine received, were recorded. Both vaccinated and unvaccinated patients were included in the study. Patients with a complete vaccination schedule were considered vaccinated according to the following criteria: for BNT162b2, mRNA-1273 and ChAdOx-1S vaccines, two doses and a minimum period of 14 days after the second dose; for Ad.26.COV.3 vaccine, a single dose and a minimum period of 14 days before infection. Patients with an incomplete vaccination schedule (n = 5) were excluded from the study.

**Figure 1:**
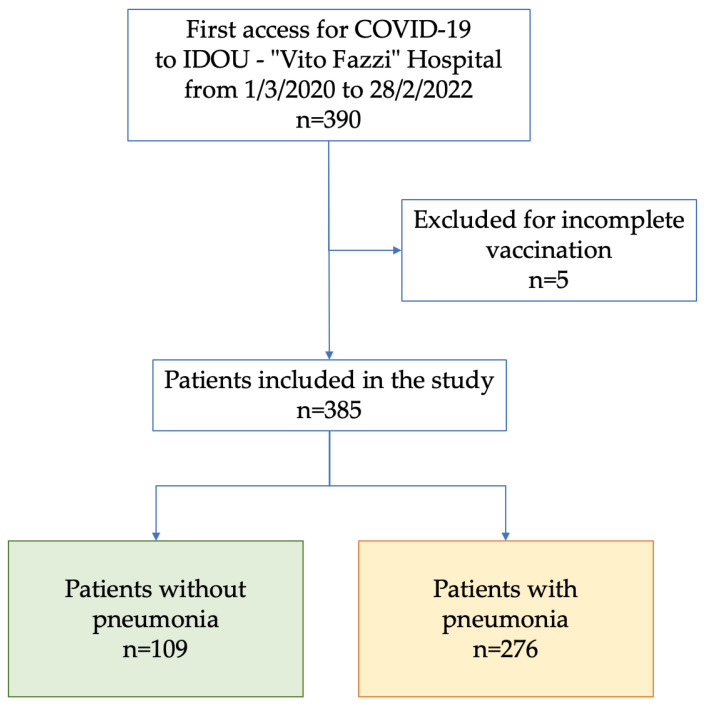
Study cohort composition (IDOU, infectious diseases operating unit).

Evidence of pneumonia at admission was detected through clinical signs and confirmed by chest radiography or High-Resolution Computed Tomography (HRCT) scan. HRCT imaging findings were classified by radiologists as ground-glass opacities (GGO), pulmonary considerations, or a mixed pattern. Based on the diagnosis of pneumonia, the subjects included in the cohort were divided into two groups: patients without pneumonia (n = 171) and patients with pneumonia (n = 276) (Figure 1).

Obesity identified by BMI ≥ 30, type 2 diabetes, hypertension under pharmacological treatment, nephropathy requiring dialysis, and cancer were considered comorbidities at admission.

In addition, laboratory data, length of hospital stay (LoS), admission to intensive care unit (ICU), and outcome of COVID-19 were collected from the patients’ medical records. For each patient, data on C-reactive protein (CRP), interleuchin-6 (IL-6), D-dimer, and glomerular filter rate (GFR) were reported. All the analyses were performed at the Central Laboratory of the Hospital using commercial kits.

### 2.2. Statistical Analysis

All data relating to individual and clinical variables were entered into a Microsoft Excel database and statistically processed using the MedCalc Software, version 14.8.1 (MedCalc Sofware Ltd., Ostend, Belgium).

The characteristics of each group of patients were summarized by means of a descriptive statistic. The categorical variables were expressed as absolute (n) and relative (%) frequency, while the comparison between groups was performed using the chi-square test. For the quantitative variables, the D’Agostino–Pearson test was first conducted to assess the distribution of values. All the continuous variables did not have a normal distribution of values; therefore, they were expressed as median and interquartile range (IQR), while the comparison between groups was carried out using the Mann–Whitney test. In all cases, differences were considered significant at *p* < 0.05.

A multivariate logistic regression analysis was performed in order to determine whether individual or clinical factors (n = 8), including age ≥ 50 years, male gender, vaccination against SARS-CoV2, obesity, diabetes, nephropathy (on dialysis), hypertension, and cancer (independent variables), were associated with pneumonia (dependent variable). Based on the number of patients included in each group, the actual EPV ratio was 13.6, indicating an adequate sample size. A *p*-value < 0.05 was set to detect the independent variables associated significantly with dependent variables. An odds ratio (OR) and 95% confidence interval (CI) were calculated and showed by a forest plot. Finally, the goodness-of-fit of logistic models was assessed using the Hosmer–Lesmeshow test with 10 subgroups based on risk deciles. Moreover, the area under the ROC curve (AUC) with 95% CI was reported to describe the predictive accuracy of the logistic model.

### 2.3. Ethical Aspects

The study was conducted according to the Declaration of Helsinki. Ethical approval was obtained by the Ethics Committee of the Health Local Unit of Lecce (Report n.57 of 22 January 2021).

## 3. Results

The study cohort included a total of 385 patients. Table 1 shows the individual and clinical features of the study group. The subjects had a mean age of 69.0 ± 16.0 years (range 18–99 years) and 59.0% of them were males. Vaccination was previously received by 76 (19.7%) patients with a higher prevalence of BNT162b2 vaccine (15.0%).

At hospital admission, 318 patients (82.6%) reported one or more comorbidity, namely 201 (52.2%) subjects were affected by hypertension, 98 (25.5%) type 2 diabetes, 84 (21.8%) obesity, 36 (9.4%) cancer, and 14 (3.6%) suffered from kidney disease and were being treated with dialysis.

Pneumonia was identified in 276 (71.7%) patients. Among the admitted patients, 92 (23.9%) were subsequently transferred to the ICU and 81 (21.0%) died. Overall, the mean LoS was 18.4 ± 11.8 days.

Table 2 shows the individual and clinical characteristics of patients without and with pneumonia. The variables were grouped as possible factors influencing the disease and possible effects of the disease. Some variables resulted in being significantly different (*p* < 0.05) between groups. Among the possible factors influencing the disease, the percentage of vaccinated patients (28.4%) was higher in the un-pneumonia group, while the prevalence of obesity (25.7%) and nephropathy on dialysis (7.9%) was higher in the pneumonia group. Among the possible effects of the disease, the PO_2_/FiO_2_ resulted in being lower (median: 275 mmHg; IQR: 199–343 mmHg) in the pneumonia group while IL-6 (median: 25.6 pg/mL; IQR: 8.1–78.3 pg/mL), CRP (median: 53.3 mg/dL; IQR: 23.7–103.4 mg/dL), admission to ICU (31.5%), and death (25.7%) were higher in the pneumonia group.

Regarding the clinical characteristics of patients as a function of vaccination status (Table 3), the prevalence of pneumonia resulted in being significantly higher (74.8%) among unvaccinated subjects, while the value of IL-6 (median: 34.5 pg/mL; IQR: 14.7–79.2 pg/mL) was found to be higher in the vaccinated patient group.

**Figure 2:**
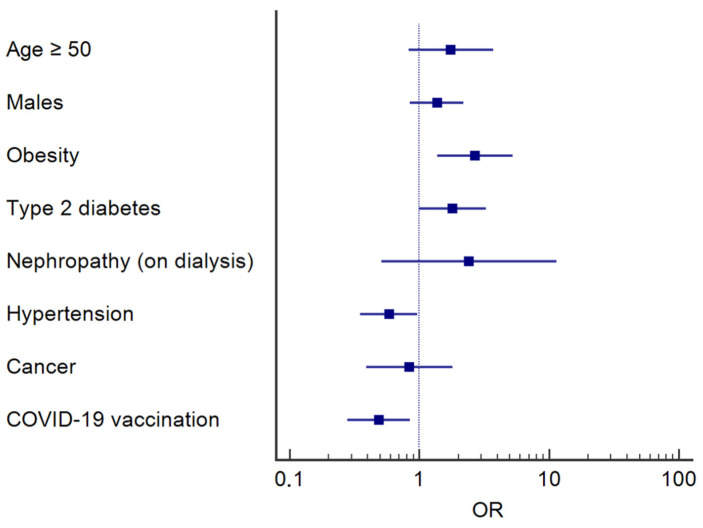
Multivariate logistic regression between pneumonia (dependent variable) and individual/clinical characteristics (independent variables) of subjects included in the study.

Multivariate regression analysis showed that pneumonia in COVID-19 patients was positively associated with obesity (OR 2.52; 95% CI 1.27–4.98) and type 2 diabetes (OR 1.81; 95% CI 1.00–3.27), and negatively with hypertension (OR 0.58; 95% CI 0.35–0.96). Vaccination against SARS-CoV-2 appears to be protective against pneumonia in COVID-19 patients (OR 0.49; 95% CI 0.28–0.85) (Figure 2). The Hosmer and Lemeshow test confirmed the goodness of fit of the logistic model with a *p*-value of 0.088. Moreover, AUC was 0.655 and the 95% CI was 0.606–0.703.

## 4. Discussion

The pandemic caused by SARS-CoV-2 has imposed a great challenge in epidemiological, diagnostic, therapeutic, and preventive research.

After three years, following the decrease in deaths and hospitalizations related to COVID-19 in the ICU as well as the high levels of immunity of the population achieved, the Emergency Committee on the COVID-19 pandemic declared that the pandemic is no longer a public health emergency of international concern and can be managed in the long term [32].

Despite the epidemiological evidence, many uncertainties remain about the future course of SARS-CoV-2 infection. Therefore, it is necessary to investigate some pathogenetic aspects through analysis of available clinical data to prevent the serious consequences of the disease in the event of further circulation of the virus. Conducting epidemiological studies to understand the factors associated with the development of a severe form of the disease will afford a better management of the disease on the basis of individual characteristics.

Our study allowed us to verify that some individual or clinical characteristics appear different on the basis of the diagnosis of pneumonia in a cohort of 385 patients aged 18 to 99 years hospitalized with COVID-19 during the pandemic.

In the whole cohort, 276 patients (71.7%) had received a diagnosis of pneumonia. The significantly different risk factors among the groups of patients without and with pneumonia were vaccination as well as some comorbidities, such as obesity and nephropathy on dialysis. Among the possible effects of the disease, the levels of PO_2_/FiO_2_ resulted in being significantly lower in the pneumonia group, while IL-6, CRP, rate of admission to ICU, and death were higher among the patients with pneumonia.

The higher levels of IL-6 and CRP observed in patients with pneumonia compared with the other group may be associated with the release of inflammatory mediators from type 2 pneumocytes as the virus enters cells via the ACE2 receptor and undergoes replication [33]. Pro-inflammatory cytokines release appears to contribute to SARS-CoV-2 lung inflammation and extensive lung injury [34]. Our data confirm a reduction in the respiratory index PO_2_/FiO_2_ and a concomitant increase in IL-6 and CRP levels, a peculiar consequence of the cytokine storm, which are significative indicators for severe COVID-19 [35].

Finally, the higher prevalence of admission to ICU and a fatal outcome among patients with pneumonia was in accordance with previously reported data [36,37].

Multivariate logistic regression analysis revealed that pneumonia in COVID-19 patients was positively associated with obesity and type 2 diabetes, and negatively with hypertension. Vaccination against SARS-CoV-2 resulted in being protective against the development of pneumonia in COVID-19 patients.

In the literature, obesity and type 2 diabetes are among the most common comorbidities in patients hospitalized with COVID-19 [38]. Patients with obesity and type 2 diabetes are physiologically fragile individuals exposed to an increased risk of recurrent infections, which can degenerate into sepsis and death [39]. Both pathologies produce a state of chronic low-grade inflammation that could facilitate the typical cytokine storm of COVID-19, contributing to amplifying its effects [40].

Obesity, here defined as BMI ≥ 30 kg/m^2^ according to the WHO’s recommendations [41], was more prevalent in the pneumonia group. As recently reported, as well as being a risk factor for several chronic and degenerative diseases, obesity increases the susceptibility to respiratory infections and has severe physiological and mechanical effects on the airways, resulting in decreased lung volume, altered ventilation patterns, and impaired overall respiratory system compliance. The overall impact of obesity on lung function is multifactorial and increases the likelihood that the obese will experience respiratory symptoms and progress to respiratory failure [42]. Furthermore, obese individuals can have chronic hormonal dysfunctions that contribute to the dysregulation of the immune response, increasing the concentration of several pro-inflammatory cytokines and leading to innate immunity deficiency. In particular, patients with obesity show the overexpression of ACE2 and other SARS-CoV-2 receptors, which results in increased viral shedding, immune inactivation, and cytokine storm [43,44]. As found in a recent study which analyzed several meta-analyses, individuals with obesity infected by SARS-CoV-2 were more likely to fall ill, to develop a more serious disease so as to require hospitalization even in intensive care, and to experience lethal endpoints [45].

Moreover, previous studies highlighted a more severe course of COVID-19 also in patients with type 2 diabetes mellitus [46,47]. Diabetes and hyperglycemia are considered independent predictors of adverse outcomes for COVID-19 [48]. Non-specific and fast-acting innate immune defenses are impaired in diabetic patients [40]. Furthermore, several studies have demonstrated that these types of patients suffer an associated pulmonary dysfunction, with physiological and structural abnormalities in the lung tissues [49]. Contrary to what occurs in obesity, a reduced expression of ACE2 in these patients, probably due to glycosylation, was demonstrated. Since ACE2 has been shown to play protective, anti-inflammatory, and antioxidant roles for lung membranes, its under expression may lead to severe lung injury and ARDS in patients with type 2 diabetes mellitus affected by COVID-19 [50]. Although it is counted among the main risk factors, the results of studies to date are somewhat mixed. Some comparative studies showed no direct correlation between diabetes and mortality for COVID-19, but rather with the variety of disorders that diabetes causes in the organism [50,51,52].

Our data show that vaccination against SARS-CoV-2 is negatively associated with pneumonia, resulting in being strongly protective against the development of severe forms of COVID-19. Different types of vaccines have been developed to prevent SARS-CoV-2 infection and subsequent COVID-19 disease [12]. Numerous studies found, as a result of vaccine administration, a lower incidence of hospitalization for COVID-19 pneumonia, fewer hospitalized patients with respiratory distress and requiring supplemental oxygen, as well as reduced need for ICU admission [53,54,55]. In particular, a recent comparative analysis of clinical outcomes conducted in Spain on 232 adult patients hospitalized with COVID-19 showed that a complete vaccination schedule of either mRNA or adenovirus vaccines protected patients from progression to severe disease or death compared to unvaccinated patients [56].

The use of the vaccine in record time has prompted the scientific community to evaluate the effectiveness of mass COVID-19 vaccination in different populations and subgroups to specify the impact of vaccines in real situations in terms of incidence of infection, mortality, and hospitalization [57]. A recent meta-analysis indicated that vaccination had a positive effect in terms of reducing all three of these effects of the virus [58].

Considering both the various vaccines available and the different variants of the virus, several authors have underlined the efficacy of complete vaccination against COVID-19 in reducing the hospitalization rate, validating the need for additional booster doses [59,60].

A metanalysis including 7 studies with a total of 1,366,700 participants (689,967 vaccinated and 676,733 non-vaccinated) reported that all types of vaccines effectively prevent the risk of severe illness after diagnosis and concluded that improving vaccine protection could reduce COVID-19-related deaths worldwide [61].

Vaccination against SARS-CoV-2 remains the primary means of limiting the spread of the virus and its effects. However, in addition to periodic booster doses in response to new variants of the virus, antiviral therapy is considered a valid therapeutic solution for frail subjects [62].

Recent studies have evaluated the safety profile and efficacy of antiviral drugs, highlighting that especially in groups at the highest risk of severe outcomes—such as the elderly and those with multiple underlying health conditions—these therapies significantly reduce hospitalization and the rate of mortality [62,63].

Our results revealed hypertension as a factor negatively associated with severe forms of the disease. This result is not surprising since all hypertensive patients in the present cohort were receiving therapy with angiotensin-converting enzyme inhibitors. A recent study on the use of ACE inhibitors for hypertensive patients with COVID-19 infection showed the protective effects of these drugs on the severity of the disease in terms of mortality, highlighting that in this group of patients, there was less disease severity and a reduced risk of admission to intensive care [64].

Our study had some limitations. First, this was a single-center study for which the results could only be extended to the reference population (the province of Lecce). In addition, due to the retrospective nature of the survey, some variables related to the patients, such as behavioral factors, other comorbidities, or further outcomes, were not available. Nor did we assess some potential confounding factors, such as previous SARS-CoV-2 infection or time between the data of vaccination and the onset of disease. Moreover, it would have been interesting to consider the effects of the restrictive measures, the risk management, and the communication plans implemented by the government during the pandemic [15,16,17,18,19,20,21,22,23,24,25,26,27,28,29,30,31,32,33,34,35,36,37,38,39,40,41,42,43,44,45,46,47,48,49,50,51,52,53,54,55,56,57,58,59,60,61,62,63,64,65].

Despite the limitations, our analysis has some strengths. Inclusion in the study cohort took place according to the principle of random sampling since it included all subjects who, during the study period, had been hospitalized in the infectious diseases operating unit of the reference center for COVID-19 in the province of Lecce. This aspect, together with the suitable sample size, allowed us to carry out an accurate analysis of the data and obtain significant results referring to the population living in the province of Lecce.

## 5. Conclusions

In the light of our findings, the presence of some comorbidities, such as obesity and type 2 diabetes, are correlated with a higher risk of developing pneumonia in COVID-19 patients. On the contrary, vaccination resulted in being a factor strongly protective against the development of pneumonia. Therefore, the results of this study support the thesis that the use of vaccines against SARS-CoV-2 prevents the severe effects of the disease, in particular in individuals with comorbidities. Furthermore, as a result of the negative correlation found between hypertension and severe forms of the disease, it could be useful to explore the protective effect of antihypertensive therapies by evaluating in future studies the effects of different types of drugs.

Finally, the results of the present study could be used in further metanalyses in order to globally evaluate the role of any factors, including vaccination, in the pathogenesis of COVID-19.

## Figures and Tables

**Table 1:** General characteristics of the subjects included in the study.

Characteristics	Patients n = 385
Gender (male), n (%)	227 (59.0)
Age (years), mean (SD)	69.0 (16.0)
COVID-19 vaccination, n (%)	76 (19.7)
BNT162b2, n (%)	58 (15.0)
mRNA-1273, n (%)	10 (2.6)
Ad.26.COV.3, n (%)	5 (1.3)
ChAdOx-1S, n (%)	3 (0.8)
Comorbidity at admission, n (%)	318 (82.6)
Hypertension, n (%)	201 (52.2)
Type 2 diabetes, n (%)	98 (25.5)
Obesity, n (%)	84 (21.8)
Cancer, n (%)	36 (9.4)
Nephropathy (on dialysis), n (%)	14 (3.6)
Pneumonia, n (%)	276 (71.7)
ICU, n (%)	92 (23.9)
Deaths, n (%)	81 (21.0)
LoS, mean (SD)	18.4 (11.8)

SD, standard deviation; LoS, length of hospital stay; ICU, intensive care unit.

**Table 2:** Individual and clinical characteristics of patients without or with pneumonia.

Variables	Un-Pneumonia n = 109	Pneumonia n = 276	*p*-Value
**Possible factors influencing the disease**			
Age ≥ 50 years, n (%)	95 (87.2)	247 (87.2)	0.634 *
Gender (male), n (%)	61 (56.0)	166 (60.1)	0.525 *
COVID-19 vaccination, n (%)	31 (28.4)	45 (16.3)	0.011 *
No. of comorbidities			
None, n (%)	19 (17.4)	48 (17.4)	
1, n (%)	25 (22.9)	66 (23.9)	
2, n (%)	32 (29.4)	72 (26.1)	
≥3, n (%)	33 (30.3)	90 (32.6)	0.907 *
Comorbidities			
Hypertension, n (%)	62 (56.9)	139 (50.4)	0.053 *
Type 2 diabetes, n (%)	20 (18.3)	78 (28.3)	0.059 *
Obesity, n (%)	13 (11.9)	71 (25.7)	0.005 *
Cancer, n (%)	13 (11.9)	23 (8.3)	0.370 *
Nephropathy (on dialysis), n (%)	2 (1.8)	12 (4.4)	0.045 *
**Possible effects of the disease**			
PO_2_/FiO_2_ (mmHg), median [IQR]	361 [300–430]	275 [199–343]	**<0.001** **
IL-6 (pg/mL), median [IQR]	14.5 [5.4–32.5]	25.6 [8.1–78.3]	**<0.001** **
D-Dimer (μg/mL), median [IQR]	1032 [556–2959]	1099 [622–2783]	0.739 **
CRP (mg/dL), median [IQR]	27.1 [6.6–56.3]	53.3 [23.7–103.4]	**<0.001** **
GFR (mL/min), median [IQR]	72.0 [52.0–85.0]	70.0 [47.5–87.0]	0.864 **
ICU, n (%)	5 (4.6)	87 (31.5)	0.001 *
Death, n (%)	10 (9.2)	71 (25.7)	0.001 *
LoS, median [IQR]	15 [10–21]	16 [11–23]	0.515 **

LoS, length of hospital stay; ICU, intensive care unit; PO_2_, partial pressure of oxygen; FiO_2_, fraction of inspired oxygen; IL-6, interleukin 6; CRP, C-reactive protein; GFR, glomerular filtration rate; * Chi-square test; ** Mann–Whitney test.

**Table 3:** Clinical characteristics of unvaccinated and vaccinated patients.

Variables	Unvaccinated n = 309	Vaccinated n = 76	*p*-Value
Pneumonia, n (%)	231 (74.8)	45 (59.2)	0.011 *
PO_2_/FiO_2_ (mmHg), median [IQR]	300 [220–364]	312 [251–405]	0.108 **
IL-6 (pg/mL), median [IQR]	18.4 [6.8–57.8]	34.5 [14.7–79.2]	0.006 **
D-Dimer (μg/mL), median [IQR]	1000 [548–2936]	1362 [853–2489]	0.057 **
CRP (mg/dL), median [IQR]	46.6 [15.9–91.1]	43.3 [18.4–114.6]	0.471 **
GFR (mL/min), median [IQR]	70.0 [48.0–86.0]	75.0 [50.0–86.0]	0.743 **
ICU, n (%)	76 (24.6)	16 (21.1)	0.618 *
Death, n (%)	62 (20.1)	19 (25.0)	0.430 *
LoS, median [IQR]	15 [11,12,13,14,15,16,17,18,19,20,21,22]	17 [10.5–25.5]	0.341 **

SD, standard deviation; LoS, length of hospital stay; ICU, intensive care unit; PO_2_, partial pressure of oxygen; FiO_2_, fraction of inspired oxygen; IL-6, interleukin 6; CRP, C-reactive protein; GFR, glomerular filtration rate; * Chi-square test; ** Mann–Whitney test.

## Data Availability

The data presented in this study are available on request from the corresponding author.

## References

[1] Adil M.T., Rahman R., Whitelaw D., Jain V., Al-Taan O., Rashid F., Munasinghe A., Jambulingam P. (2021). SARS-CoV-2 and the pandemic of COVID-19. Postgrad. Med. J..

[2] Muralidar S., Ambi S.V., Sekaran S., Krishnan U.M. (2020). The emergence of COVID-19 as a global pandemic: Understanding the epidemiology, immune response and potential therapeutic targets of SARS-CoV-2. Biochimie.

[3] Rathi H., Burman V., Datta S.K., Rana S.V., Mirza A.A., Saha S., Kumar R., Naithani M. (2021). Review on COVID-19 Etiopathogenesis, Clinical Presentation and Treatment Available with Emphasis on ACE2. Indian J. Clin. Biochem..

[4] Goyal D., Inada-Kim M., Mansab F., Iqbal A., McKinstry B., Naasan A.P., Millar C., Thomas S., Bhatti S., Lasserson D. (2021). Improving the early identification of COVID-19 pneumonia: A narrative review. BMJ Open Respir. Res..

[5] Zizza A., Recchia V., Aloisi A., Guido M. (2021). Clinical features of COVID-19 and SARS epidemics. A literature review. J. Prev. Med. Hyg..

[6] De Donno A., Lobreglio G., Panico A., Grassi T., Bagordo F., Bozzetti M.P., Massari S., Siculella L., Damiano F., Guerra F. (2021). IgM and IgG Profiles Reveal Peculiar Features of Humoral Immunity Response to SARS-CoV-2 Infection. Int. J. Environ. Res. Public Health.

[7] Long B., Carius B.M., Chavez S., Liang S.Y., Brady W.J., Koyfman A., Gottlieb M. (2022). Clinical update on COVID19 for the emergency clinician: Presentation and evaluation. Am. J. Emerg. Med..

[8] Wang D., Hu B., Hu C., Zhu F., Liu X., Zhang J., Wang B., Xiang H., Cheng Z., Xiong Y. (2020). Clinical Characteristics of 138 Hospitalized Patients with 2019 Novel Coronavirus-Infected Pneumonia in Wuhan, China. JAMA.

[9] World Health Organization (WHO) (2021). Coronavirus Disease (COVID-19) Weekly Epidemiological Update and Weekly Operational Update. https://www.who.int/emergencies/diseases/novel-coronavirus-2019/situation-reports.

[10] Barouch D.H., Stephenson K.E., Sadoff J., Yu J., Chang A., Gebre M., McMahan K., Liu J., Chandrashekar A., Patel S. (2021). Durable Humoral and Cellular Immune Responses 8 Months after Ad26.COV2.S Vaccination. N. Engl. J. Med..

[11] Nyberg T., Ferguson N.M., Nash S.G., Webster H.H., Flaxman S., Andrews N., Hinsley W., Bernal J.L., Kall M., Bhatt S. (2022). Comparative analysis of the risks of hospitalisation and death associated with SARS-CoV-2 omicron (B.1.1.529) and delta (B.1.617.2) variants in England: A cohort study. Lancet.

[12] Forni G., Mantovani A. (2021). COVID-19 Commission of Accademia Nazionale dei Lincei, Rome. COVID-19 vaccines: Where we stand and challenges ahead. Cell. Death Differ..

[13] Panico A., Lobreglio G., Bagordo F., Zizza A., De Donno A., Rosato C., Lazzari R., Chicone M., Indino F., Recchia V. (2022). Antibody Response in Healthcare Workers before and after the Third Dose of Anti-SARS-CoV-2 Vaccine: A Pilot Study. Vaccines.

[14] Grassi T., Lobreglio G., Panico A., Rosato C., Zizza A., Lazzari R., Chicone M., Indino F., Bagordo F. (2022). Kinetics of Humoral Immunity against SARS-CoV-2 in Healthcare Workers after the Third Dose of BNT162b2 mRNA Vaccine. Vaccines.

[15] Bartolomeo N., Giotta M., Trerotoli P. (2021). In-Hospital Mortality in Non-COVID-19-Related Diseases before and during the Pandemic: A Regional Retrospective Study. Int. J. Environ. Res. Public Health.

[16] Banchelli F., Negro P., Guido M., D’Amico R., Fittipaldo V.A., Grima P., Zizza A. (2022). The Role of ABO Blood Type in Patients with SARS-CoV-2 Infection: A Systematic Review. J. Clin. Med..

[17] Damiani A.S., Zizza A., Banchelli F., Gigante M., De Feo M.L., Ostuni A., Marinelli V., Quagnano S., Negro P., Di Renzo N. (2023). Association between ABO blood groups and SARS-CoV-2 infection in blood donors of Puglia region. Ann. Hemato..

[18] Grima P., Guido M., Zizza A. (2023). Clinical characteristics and risk factors associated with COVID-19 mortality in a non-Intensive Care Unit. J. Prev. Med. Hyg..

[19] Meister T., Pisarev H., Kolde R., Kalda R., Suija K., Milani L., Karo-Astover L., Piirsoo M., Uusküla A. (2022). Clinical characteristics and risk factors for COVID-19 infection and disease severity: A nationwide observational study in Estonia. PLoS ONE.

[20] Negro P., Congedo M., Zizza A., Guido M., Sacquegna G., Pulito G., Lobreglio G. (2022). Role of ABO blood system in COVID-19: Findings from a southern Italian study. Transfus. Med..

[21] Sanyaolu A., Okorie C., Marinkovic A., Patidar R., Younis K., Desai P., Hosein Z., Padda I., Mangat J., Altaf M. (2020). Comorbidity and its Impact on Patients with COVID-19. SN Compr. Clin. Med..

[22] Vadgama N., Kreymerman A., Campbell J., Shamardina O., Brugger C., Deaconescu A.M., Lee R.T., Penkett C.J., Gifford C.A., Genomic England Research Consortium (2022). SARS-CoV-2 Susceptibility and ACE2 Gene Variations within Diverse Ethnic Backgrounds. Front. Genet..

[23] Fauci A.S., Lane H.C., Redfield R.R. (2020). COVID-19—Navigating the uncharted. N. Eng. J. Med..

[24] Mulchandani R., Lyngdoh T., Kakkar A.K. (2021). Deciphering the COVID-19 cytokine storm: Systematic review and meta-analysis. Eur. J. Clin. Investig..

[25] Trofin F., Nastase E.V., Roșu M.F., Bădescu A.C., Buzilă E.R., Miftode E.G., Manciuc D.C., Dorneanu O.S. (2023). Inflammatory Response in COVID-19 Depending on the Severity of the Disease and the Vaccination Status. Int. J. Mol. Sci..

[26] Bernard G.R., Artigas A., Brigham K.L., Carlet J., Falke K., Hudson L., Lamy M., Legall J.R., Morris A., Spragg R. (1994). The American European Consensus Conference on ARDS. Definitions, mechanisms, relevant outcomes, and clinical trial coordination. Am. J. Respir. Crit. Care Med..

[27] Zheng C., Shao W., Chen X., Zhang B., Wang G., Zhang W. (2022). Real-world effectiveness of COVID-19 vaccines: A literature review and meta-analysis. Int. J. Infect. Dis..

[28] Keswani A., Dhana K., Rosenthal J.A., Moore D., Mahdavinia M. (2020). Atopy is predictive of a decreased need for hospitalization for coronavirus disease 2019. Ann. Allergy Asthma Immunol..

[29] Shakoor H., Feehan J., Al Dhaheri A.S., Ali H.I., Platat C., Ismail L.C., Apostolopoulos V., Stojanovska L. (2021). Immune-boosting role of vitamins D, C, E, zinc, selenium and omega-3 fatty acids: Could they help against COVID-19?. Maturitas.

[30] Istituto Nazionale di Statistica (ISTAT) Popolazione Residente al 1° Gennaio. http://dati.istat.it/Index.aspx?DataSetCode=DCIS_POPRES1#.

[31] Moons K.G., Altman D.G., Reitsma J.B., Ioannidis J.P., Macaskill P., Steyerberg E.W., Vickers A.J., Ransohoff D.F., Collins G.S. (2015). Transparent Reporting of a multivariable prediction model for Individual Prognosis or Diagnosis (TRIPOD): Explanation and elaboration. Ann. Intern. Med..

[32] World Health Organization (WHO) (2023). Statement on the Fifteenth Meeting of the IHR (2005) Emergency Committee on the COVID-19 Pandemic. https://www.who.int/news/item/05-05-2023-statement-on-the-fifteenth-meeting-of-the-international-health-regulations-.

[33] Chi Y., Ge Y., Wu B., Zhang W., Wu T., Wen T., Liu J., Guo X., Huang C., Jiao Y. (2020). Serum cytokine and chemokine profile in relation to the severity of coronavirus disease 2019 in China. J. Infect. Dis..

[34] Iwamura A.P.D., Tavares da Silva M.R., Hümmelgen A.L., Soeiro Pereira P.V., Falcai A., Grumach A.S., Goudouris E., Neto A.C., Prando C. (2021). Immunity and inflammatory biomarkers in COVID-19: A systematic review. Rev. Med. Virol..

[35] Coperchini F., Chiovato L., Croce L., Magri F., Rotondi M. (2020). The cytokine storm in COVID-19: An overview of the involvement of the chemokine/chemokine-receptor system. Cytokine Growth Factor Rev..

[36] Huang C., Wang Y., Li X., Ren L., Zhao J., Hu Y., Zhang L., Fan G., Xu J., Gu X. (2020). Clinical features of patients infected with 2019 novel coronavirus in Wuhan, China. Lancet.

[37] Yang X., Yu Y., Xu J., Shu H., Xia J., Liu H., Wu Y., Zhang L., Yu Z., Fang M. (2020). Clinical course and outcomes of critically ill patients with SARS-CoV-2 pneumonia in Wuhan, China: A single-centered, retrospective, observational study. Lancet Respir. Med..

[38] Javid F.A., Waheed F.A., Zainab N., Khan H., Amin I., Bham A., Ghoghawala M., Sheraz A., Haloub R. (2023). COVID-19 and diabetes in 2020: A systematic review. J. Pharm. Policy Pract..

[39] Frydrych L.M., Bian G., O’Lone D.E., Ward P.A., Delano M.J. (2018). Obesity and type 2 diabetes mellitus drive immune dysfunction, infection development, and sepsis mortality. J. Leukoc. Biol..

[40] Zhou Y., Chi J., Lv W., Wang Y. (2021). Obesity and diabetes as high-risk factors for severe coronavirus disease 2019 (COVID-19). Diabetes Metab. Res. Rev..

[41] Jensen M.D., Ryan D.H., Apovian C.M., Ard J.D., Comuzzie A.G., Donato K.A., Hu F.B., Hubbard V.S., Jakicic J.M., Kushner R.F. (2014). 2013 AHA/ACC/TOS guideline for the management of overweight and obesity in adults: A report of the American College of Cardiology/American Heart Association Task Force on Practice Guidelines and The Obesity Society. J. Am. Coll. Cardiol..

[42] Dixon A.E., Peters U. (2018). The effect of obesity on lung function. Expert. Rev. Respir. Med..

[43] Zhang Y., Somers K.R., Becari C., Polonis K., Pfeifer M.A., Allen A.M., Kellogg T.A., Covassin N., Singh P. (2018). Comparative expression of renin-angiotensin pathway proteins in visceral versus subcutaneous fat. Front. Physiol..

[44] Ryan P.M., Caplice N.M. (2020). Is adipose tissue a reservoir for viral spread, immune activation and cytokine amplification in COVID-19. Obesity.

[45] Popkin B.M., Du S., Green W.D., Beck M.A., Algaith T., Herbst C.H., Alsukait R.F., Alluhidan M., Alazemi N., Shekar M. (2021). Individuals with obesity and COVID-19: A global perspective on the epidemiology and biological relationships. Obes. Rev..

[46] Huang I., Lim M.A., Pranata R. (2020). Diabetes mellitus is associated with increased mortality and severity of disease in COVID-19 pneumonia—A systematic review, meta-analysis, and meta-regression. Diabetes Metab. Syndr..

[47] Al-Salameh A., Lanoix J.P., Bennis Y., Andrejak C., Brochot E., Deschasse G., Dupont H., Goeb V., Jaureguy M., Lion S. (2021). Characteristics and outcomes of COVID-19 in hospitalized patients with and without diabetes. Diabetes Metab. Res. Rev..

[48] Long H., Li J., Li R., Zhang H., Ge H., Zeng H., Chen X., Lu Q., Jiang W., Zeng H. (2022). Plasma glucose levels and diabetes are independent predictors for mortality in patients with COVID-19. Epidemiol. Infect..

[49] Teeter J.G., Riese R.J. (2008). Cross-sectional and prospective study of lung function in adults with type 2 diabetes: The Atherosclerosis Risk in Communities (ARIC) study: Response to Yeh et al. Diabetes Care.

[50] Pal R., Bhansali A. (2020). COVID-19, diabetes mellitus and ACE2: The conundrum. Diabetes Res. Clin. Pract..

[51] Said K.B., Alsolami A., Alreshidi F.S., Fathuddin A., Alshammari F., Alrashid F., Aljadani A., Aboras R., Alreshidi F., Alghozwi M.H. (2023). Profiles of Independent-Comorbidity Groups in Senior COVID-19 Patients Reveal Low Fatality Associated with Standard Care and Low-Dose Hydroxychloroquine over Antivirals. J. Multidiscip. Health.

[52] Alguwaihes A.M., Al-Sofiani M.E., Megdad M., Albader S.S., Alsari M.H., Alelayan A., Alzahrani S.H., Sabico S., Al-Daghri N.M., Jammah A.A. (2020). Diabetes and Covid-19 among hospitalized patients in Saudi Arabia: A single-centre retrospective study. Cardiovasc. Diabetol..

[53] Cianci R., Franza L., Pignataro G., Massaro M.G., Rio P., Tota A., Ocarino F., Sacco Fernandez M., Franceschi F., Gasbarrini A. (2023). Effect of COVID-19 Vaccination on the In-Hospital Prognosis of Patients Admitted during Delta and Omicron Waves in Italy. Vaccines.

[54] Kelly J.D., Leonard S., Hoggatt K.J., Boscardin W.J., Lum E.N., Moss-Vazquez T.A., Andino R., Wong J.K., Byers A., Bravata D.M. (2022). Incidence of Severe COVID-19 Illness Following Vaccination and Booster with BNT162b2, mRNA-1273, and Ad26.COV2.S Vaccines. JAMA.

[55] Bagshaw S.M., Abbott A., Beesoon S., Bowker S.L., Zuege D.J., Thanh N.X. (2023). A population-based assessment of avoidable hospitalizations and resource use of non-vaccinated patients with COVID-19. Can. J. Public Health.

[56] Bernal E., García-Villalba E., Pons E., Vicente M.R., Tomás C., Minguela A., GERS (2023). Role of vaccination and anti-SARS-CoV-2 antibodies in the clinical outcome of hospitalized COVID-19 patients. Med. Clin..

[57] Thompson M.G., Stenehjem E., Grannis S., Ball S.W., Naleway A.L., Ong T.C., DeSilva M.B., Natarajan K., Bozio C.H., Lewis N. (2021). Effectiveness of Covid-19 vaccines in outpatient and inpatient care settings. N. Eng. J. Med..

[58] Rahmani K., Shavaleh R., Forouhi M., Disfani H.F., Kamandi M., Oskooi R.K., Foogerdi M., Soltani M., Rahchamani M., Mohaddespour M. (2022). The efficacy of COVID-19 vaccines in reducing COVID-19 incidence, hospitalization, and mortality: A systematic review and meta-analysis. Public Health Front..

[59] Tartof S.Y., Slezak J.M., Puzniak L., Hong V., Frankland T.B., Xie F., Ackerson B.K., Valluri S.R., Jodar L., McLaughlin J.M. (2023). Efficacy and durability of the BNT162b2 vaccine against hospital and emergency room admissions due to SARS-CoV-2 omicron strains BA.1 and BA.2 in a large health care system in the United States: A test-negative case-control study. Lancet Respir. Med..

[60] Hogan A.B., Doohan P., Wu S.L., Mesa D.O., Toor J., Watson O.J., Winskill P., Charles G., Barnsley G., Riley E.M. (2023). Estimating long-term vaccine efficacy against SARS-CoV-2 variants: A model-based approach. Nat. Munic..

[61] Huang Y.Z., Kuan C.C. (2022). Vaccination to reduce severe COVID-19 and mortality in COVID-19 patients: A systematic review and meta-analysis. Eur. Rev. Med. Pharmacol. Sci..

[62] Aiello T.F., García-Vidal C., Soriano A. (2022). Antiviral drugs against SARS-CoV-2. Rev. Esp. Quimioter..

[63] Bruno G., Giotta M., Perelli S., De Vita G., Bartolomeo N., Buccoliero G.B. (2022). Early access to oral antivirals in high-risk outpatients: Good weapons to fight COVID-19. Virus.

[64] Acharya S., Kumar S., Kabra R., Patel M., Phate N., Talwar D., Daiya V. (2023). Impact of angiotensin receptor blocker as antihypertensive in assessing mortality in patients of COVID-19: A single tertiary care center study. J. Educ. Health Promot..

[65] Recchia V., Aloisi A., Zizza A. (2022). Risk management and communication plans from SARS to COVID-19 and beyond. Int. J. Health. Plann. Manag..

